# Robust high-throughput kinetic analysis of apoptosis with real-time high-content live-cell imaging

**DOI:** 10.1038/cddis.2016.332

**Published:** 2016-12-01

**Authors:** Jesse D Gelles, Jerry Edward Chipuk

**Affiliations:** 1 Department of Oncological Sciences, Icahn School of Medicine at Mount Sinai, New York, USA; 2 The Tisch Cancer Institute, Icahn School of Medicine at Mount Sinai, New York, USA; 3 The Graduate School of Biomedical Sciences, Icahn School of Medicine at Mount Sinai, New York, USA; 4 Department of Dermatology, Icahn School of Medicine at Mount Sinai, New York, USA; 5 The Diabetes, Obesity, and Metabolism Institute, Icahn School of Medicine at Mount Sinai, New York, USA

## Abstract

Quantitative and kinetic analyses of apoptotic cell death are integral components of exploring cell biology, measuring cellular stress responses, and performing high-throughput genomic/RNAi/drug screens. Here, we present a detailed method that integrates robust kinetic real-time high-content imaging with Annexin V labelling to provide a highly sensitive, accurate, simple and zero-handling approach to quantify extrinsic and intrinsic inducers of apoptosis. The sensitivity of this non-toxic method outperforms previous high-throughput methodologies using viability dyes or caspase-activation reporters. This method also incorporates a multiplex adaptation to integrate variability in cell number due to treatment-induced proliferation changes and the detachment of dying cells. Compared to Annexin V detection by flow cytometry, this method is 10-fold more sensitive, eliminates extensive sample handling and processing, and provides real-time kinetics of apoptosis at both single-cell and population-level resolutions.

The quantification of cell death responses is an integral component of exploring cell biology, responses to cellular stress and performing high-throughput drug screens. Apoptosis is the mechanism of cell death most relevant to many studies, and the gold standard method to detect apoptosis is classical Annexin V-binding assays. These assays detect early events involved in the orchestrated dismantling of apoptotic cells that proceed via the activation of caspases.^1, 2^ In survival conditions, the phospholipid phosphatidylserine (PS) is retained in the inner leaflet of the plasma membrane through lipid flippases, which are cleaved by caspases during apoptosis resulting in the stable exposure of PS in the outer leaflet of the plasma membrane.^3^ Extracellular-facing PS is recognized by Annexin V, and the stoichiometric binding is used to designate cells committed to an apoptotic programme.^4, 5^

The detection and quantification of Annexin V positive cells is usually accomplished by flow cytometry, which requires extensive sample handling, non-trivial cell numbers, and significant delays between harvest and analyses. Furthermore, as experiments must be terminated prior to analysis, flow cytometry-based Annexin V assays only provide end-point data, requiring tedious optimization for treatment, timing and harvesting. Additionally, sample preparation for flow cytometry exposes cells to mechanical and chemical stress, which results in plasma membrane instability and subsequent staining of apoptotic reporters. Collectively, these limitations hinder the depth and accuracy of collected data while burdening the investigator with labour-intensive protocols.

The recent advent of high-content live-cell imaging technologies has provided researchers with the ability to visualize cellular phenotypes in high-throughput multi-well formats. Frequently, these assays are accomplished using fluorescent reporters and analysed to provide kinetic data for the duration of the experiment. One common application of this technology is the measurement of cytotoxicity following cellular stresses, genome-wide screens and high-throughput drug screens. Unfortunately, the majority of cytotoxicity analyses are imperfect due to use of cell viability dyes (that is, propidium iodide, DRAQ7, SYTOX), which detect only late apoptotic events and do not distinguish between cell death mechanisms.^6^ Furthermore, cellular labelling with viability dyes is not stoichiometric and results in marked labelling following the first instance of membrane instability. Fluorophore-labelled caspase-cleavable probes (for example, DEVD) are also commonly utilized despite reports of differential or attenuated cleavage when compared to physiological caspase substrates as well as activation by non-caspase proteases.^7, 8^ Moreover, many laboratories employ additional secondary processing steps (for example, flow cytometry methods to count cells in each well) following the acquisition of high-content live-cell imaging data due to a lack of validated protocols controlling for inter-well plating variability and proliferation changes due to treatments. Collectively, these practices undermine the high-throughput nature of live-cell imagers and are limited by the commercially available reporters.

Here, we provide new methods, necessary controls and critical interpretations for highly sensitive Annexin V-binding assays in real-time using high-content live-cell imaging. These non-toxic methods outperform previous high-throughput methodologies and provides accurate apoptotic kinetics at both single-cell and population-level resolutions. Here we provide data using SV40-transformed mouse embryonic fibroblasts (MEFs), but have validated our methods in human, primary, transformed and cancerous cell lines. Compared to the current classical detection of Annexin V-binding by flow cytometry, our method eliminates extensive sample processing and perturbation, demonstrates greater detection sensitivity, increased accuracy of apoptotic onset and progression, provides cell phenotype data, and requires significantly less time to complete (Figure 1a).

## Results and Discussion

### Detection of Annexin V-labelled apoptotic cells in high-content live-cell imagers

**Figure 1:**
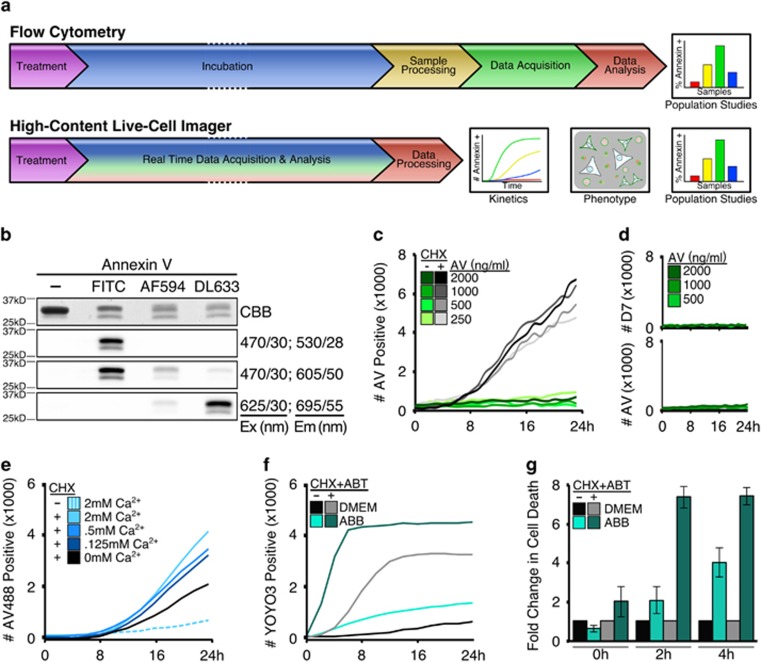
High-content live-cell imagers provide kinetic real-time Annexin V-binding data without the inherent cell toxicity compared to standard protocols. (**a**) Annexin V-binding assay workflow by either flow cytometry or high-content live-cell imaging. (**b**) Recombinant Annexin V analysed by SDS-PAGE and visualized by Coomassie brilliant blue (CBB) or indicated fluorescent filter sets; excitation and emission labelled as filter/bandpass in nm. (**c**) MEFs were plated, treated as indicated (CHX, 25 μg/ml), incubated with Annexin V-488 (indicated) in growth media, and scanned every hour for 24 h with four frames per well. Events per frame per time point were averaged. (**d**) MEFs prepared as in (c) and co-incubated with DRAQ7 (600 nM). (**e**) MEFs were treated/supplemented as indicated (CHX, 50 μg/ml) in the presence of Annexin V-488 (1 μg/ml), and scanned every 2 h for 24 h with one frame per well. Data averaged and representative of at least three experiments. (**f**) MEFs plated and scanned as in (e), incubated in YOYO3-containing (200 nM) DMEM or ABB, and treated with CHX (50 μg/ml) and ABT-737 (1 μM) to sensitize to apoptosis. (**g**) Data from (f) converted to fold increase of cytotoxicity in ABB-incubated samples compared to DMEM-incubated samples. Error bars denote standard deviation of triplicate samples

In order to determine if Annexin V could label apoptotic cells using high-content live-cell imaging, MEFs were incubated with recombinant Annexin V, treated with common inducers of apoptosis, and subjected to live-cell imaging at defined intervals. Recombinant Annexin V was labelled with either FITC (Annexin V-488, green) or AlexaFluor 594 (Annexin V-594, red) fluorophores (Figure 1b, Supplementary Figure S1A) for detection by high-content imagers as either single markers or in combination with viability dyes.^9^ MEFs treated with cycloheximide (CHX), a pro-apoptotic inhibitor of protein synthesis, labelled with Annexin in a time-dependent manner at Annexin V concentrations as low as 0.25 μg/ml (7 nM), which is approximately 10-fold less compared to concentrations used in traditional flow cytometry-based approaches (~2.5 μg/ml, 70 nM) for Annexin V-labelling (Figure 1c).

To ensure the above Annexin V-labelling occurred in response to cell death, CHX-treated MEFs were analysed following co-incubation with Annexin V and DRAQ7, a cell-impermeable viability dye commonly used in cytotoxic assays.^10^ In this assay, Annexin V-positivity markedly preceded DRAQ7-positivity, which reflects the early exposure of PS followed by the unregulated loss of plasma membrane integrity during late stages of apoptosis (Supplementary Figure S1B).^11^ In comparison, vehicle-treated cells cultured in the presence of Annexin V displayed no DRAQ7-positivity (Figure 1d) or changes in proliferation (data not shown) suggesting this method is non-toxic and well-tolerated.

The above results demonstrate that Annexin V labels apoptotic cells in culture, yet traditional Annexin V-staining protocols state a requirement for a high-calcium buffer (1.5–2 mM CaCl_2_) for Annexin V to bind exposed PS.^12^ Therefore, we investigated if additional Ca^2+^ ions enhanced Annexin V binding of apoptotic cells by supplementing CaCl_2_ to the cell media. While additional Ca^2+^ improved labelling intensity (Figure 1e, blue solid lines), it also resulted in the accumulation of Annexin V-positive puncta on cell surfaces for both Annexin V-488 and Annexin V-594 (Figure 1e, dashed line; Supplementary Figure S1C). Since Dulbecco’s modified Eagle’s medium (DMEM) contains 1.8 mM Ca^2+^ and demonstrated sufficient Annexin V labelling, the parameters for fluorescent detection of Annexin V-labelled cells were defined using signal acquired from cells in calcium-unsupplemented DMEM (refer to Materials and Methods).

Cells harvested for traditional flow cytometry-based approaches to detect Annexin V positive populations are often exposed to Annexin Binding Buffer (ABB) for several hours during staining and data acquisition.^9^ We noted that cells incubated in ABB for just a few hours demonstrated increased basal rates of apoptosis and these conditions synergized with pro-apoptotic agents (Figure 1f). For example, vehicle-treated MEFs cultured in ABB demonstrated twofold increased basal rates of apoptosis, and treatment with CHX and ABT-737 (an inhibitor of anti-apoptotic BCL-2 proteins) revealed eightfold more apoptosis compared to cells in DMEM (Figure 1g). These data suggest that quantification of Annexin V-positive cells using traditional methods is possibly obfuscated due to the synergistic stress caused by common buffers. Therefore, all subsequent experiments comparing flow cytometry and high-content live-cell imaging based methods for Annexin V-labelling were performed with DMEM and not ABB.

### Dual-reporter method for kinetic analysis of apoptotic progression

**Figure 2:**
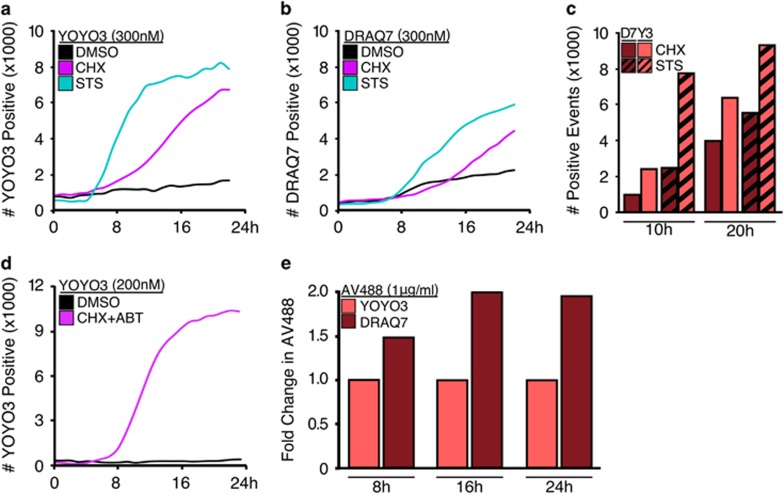
YOYO3 is a potent and non-toxic reporter of late-stage apoptosis suitable for kinetic analysis in high-content long-duration analysis. (**a**) MEFs were treated with CHX (50 μg/ml) or STS (1 μM), incubated with YOYO3 (300 nM), and scanned every 2 h for red fluorescence for 22 h. (**b**) MEFs treated and analysed as in (a) but incubated with DRAQ7 (300 nM). (**c**) Number of events detected from (a) and (b) were directly compared at 10 and 20 h. Bar graphs depict data from single experiment but representative of results from at least three experiments. (**d**) MEFs were treated with CHX (50 μg/ml) and ABT-737 (1 μM), and co-incubated with YOYO3 (200 nM), scanned every 2 h for 24 h. Data are representative of at least three experiments. (**e**) MEFs incubated with Annexin V-488 (1 μg/ml) and either YOYO3 (200 nM) or DRAQ7 (600 nM) were directly compared at several time points for relative toxicity. Data shown are from a single experiment but representative of at least three experiments

A common requirement of cell death research is the measure of early (stable exposure of PS on the extracellular leaflet of the plasma membrane) and late apoptotic events (loss of plasma membrane integrity), which in flow cytometry-based protocols is commonly accomplished by dual staining with Annexin V and propidium iodide (for example, AV^+^PI^−^ staining indicates early events while double positivity indicates late events).^13^ However, PI is incompatible with our high-content long-duration imaging assay and is toxic with prolonged exposure (data not shown). Therefore, we screened several recently developed viability dyes (DRAQ7 and YOYO3) for compatibility within our assay and no toxicity. MEFs treated with inducers of apoptosis (CHX, or the pan-kinase inhibitor staurosporine, STS) were incubated with either DRAQ7 or YOYO3. In response to the apoptotic stimuli, more cells stained faster with YOYO3 than DRAQ7 (Figures 2a and c), and YOYO3 efficiently labelled cells at lower concentrations (Figure 2d). Finally, a comparison of DRAQ7 and YOYO3 exposure from 8 to 24 h revealed a twofold increase in apoptosis as measured by Annexin V-staining (Figure 2e). Collectively, these data suggest YOYO3 is an appropriate, non-toxic viability dye suitable for prolonged incubation in this new method.

**Figure 3:**
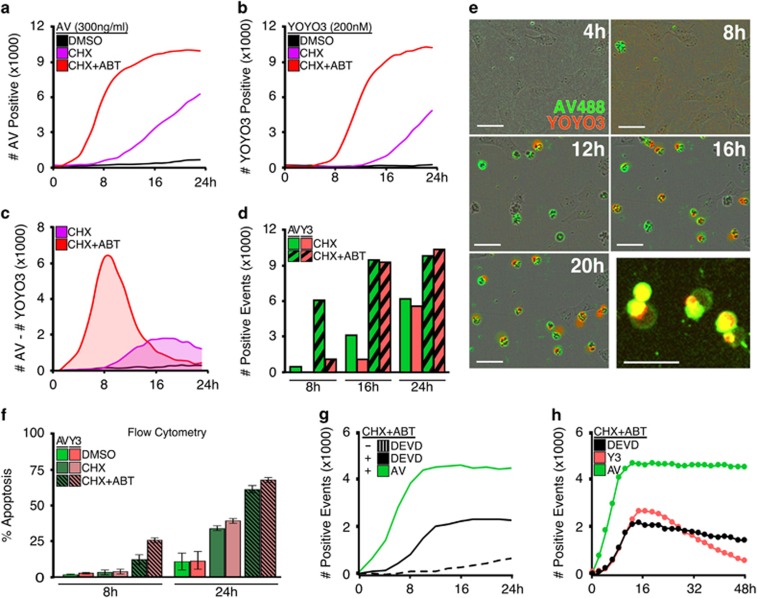
Annexin V is an easy non-toxic, and robust reporter for use in high-contrast cell imagers. (**a** and **b**) MEFs were treated with either vehicle, CHX (50 μg/ml) alone, or CHX (50 μg/ml) and ABT-737 (1 μM), co-incubated with both Annexin V-488 (1 μg/ml) and YOYO3 (200 nM), and scanned at 2-h intervals up to 24 h for green or red fluorescence, respectively. (**c**) Graph depict the difference of values collected at each time point from cells in (**a** and **b**). (**d**) Data from (**a** and **b**) represented as bar graphs for comparison between Annexin V- and YOYO3-labelling at indicated time points. (**e**) Captured images from IncuCyte Zoom using a × 10 objective for CHX-treated cells from (**a** and **b**); 50 μm scale bars. Data shown in (**a***—***e**) are from a single experiment and representative of at least three experiments. (**f**) MEFs treated as in (**a**) but analysed by flow cytometry. (**g**) MEFs treated with CHX (50 μg/ml) and ABT-737 (1 μM) were co-incubated with Annexin-594 (1 μg/ml) and CellEvent Caspase-3/-7 Green Reporter (labelled as ‘DEVD’, 2 μM), and scanned every 2 h for 24 h. (**h**) MEFs treated as in (**g**) were incubated with either Annexin-488 (1 μg/ml), YOYO3 (200 nM) or DEVD reporter (2 μM), and scanned repeatedly every 2 h for 48 h to assess photostability

Next, we integrated YOYO3 and Annexin V staining in high-content cell imaging for real-time kinetic detection of apoptosis. MEFs were treated with apoptotic stimuli in the presence of Annexin V-488 and YOYO3 and imaged every 2 h for 24 h. As shown in Figures 3a–c, Annexin V more rapidly detects apoptotic cells compared to YOYO3; similarly, YOYO3 fails to detect an apoptotic population before 8 h (Figures 3c and d). However, time points representing the onset of late apoptotic events demonstrate equivalent labelling by both Annexin V and YOYO3, further emphasizing the importance of collecting kinetic data (Figure 3d). The differences between Annexin V and YOYO3 labelling were verified by inspecting photographs captured by the high-content imager at the indicated times (Figure 3e). Interestingly, cells treated as above but subjected to preparation for flow cytometry demonstrated no differential labelling between Annexin V and YOYO3 (Figure 3f). We suspect this is due to physical stress applied during sample preparation resulting in the incorporation of YOYO3 prior to the cellular dismantling of the plasma membrane.

The majority of apoptosis detection assays require caspase activation; as such, caspase-cleavable peptide reporters (for example, the conserved caspase-3/-7 cleavage site derived from PARP, D-E-V-D) are commonly used in high-throughput assays. Next we compared the sensitivity of Annexin V to a DEVD reporter by treating MEFs with apoptotic stimuli in the presence of both detection reagents. When directly compared, Annexin V staining occurred more rapidly and on more cells than the DEVD peptide (Figure 3g). Furthermore, Annexin V demonstrated robust photostability while both the DEVD peptide and YOYO3 photobleached during data acquisition over 48 h (Figure 3h). Collectively, these data suggest that Annexin V is a superior labelling reagent for kinetic analysis. Additionally, while YOYO3 does photobleach it appropriately couples with Annexin V staining in high-content cell imaging to reveal early and late apoptotic events in one assay for both single-cell (Figure 3e) and population studies (Figure 3c).

Up to this point, we examined common inducers of apoptosis to test the validity of this methodology. Next we investigated additional treatments that engage distinct pro-apoptotic signalling mechanisms. Apoptosis occurs through two pathways: the extrinsic pathway, which is activated by extracellular ligands (for example, Tumour Necrosis Factor Alpha, TNF*α*) that bind to death receptors; and the intrinsic pathway, which is engaged by cellular stress (for example, terminal Unfolded Protein Response activated by Tunicamycin, Tn, or Thapsigargin, Tg).^14, 15, 16, 17^

**Figure 4:**
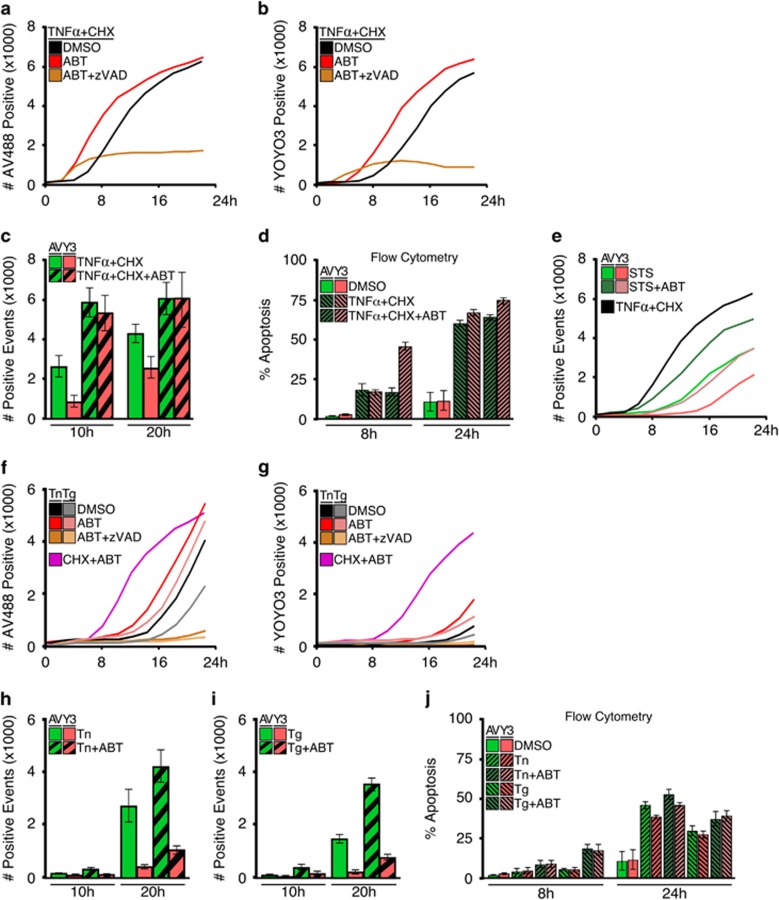
Annexin V coupled with high-content cell imaging reveals the kinetics of apoptosis activated by the extrinsic or intrinsic pathways. (**a**) MEFs were treated with TNF*α* (10 ng/ml) and CHX (50 μg/ml), co-treated with vehicle, ABT-737 (1 μM) or zVAD-fmk (100 μM), and co-incubated with Annexin V-488 (1 μg/ml) and YOYO3 (200 nM). Samples were scanned every 2 h for 22 h, for detection of green fluorescence. (**b**) MEFs treated as in (**a**) and scanned for red fluorescence. (**c**) Comparison of Annexin V-488 and YOYO3 detection of samples from (**a** and **b**) at selected time points. (**d**) MEFs treated as in (**a**) were analysed by flow cytometry in DMEM using Annexin V-488 (2 μg/ml) and YOYO3 (200 nM). (**e**) MEFs were treated with STS (1 μM)±ABT-737 (1μM) and scanned every 2 h for green and red fluorescence. TNF*α*+CHX treatment as in (**a**). (**f**) MEFs were treated with tunicamycin (Tn, 2 μg/ml) or thapsigargin (Tg, 2 μM), and co-treated and detected as in (**a**). (**g**) MEFs treated as in (**f**) were analysed for YOYO3-positivity. (**h** and **i**) Comparison of Annexin V-488 and YOYO3-positivity for samples from (**f** and **g**) at indicated times. MEFs treated as in (**f**) were analysed by flow cytometry in DMEM using Annexin V-488 (2 μg/ml) and YOYO3 (200 nM). Data shown are averages of triplicates and representative of several experiments. Error bars depict standard deviation

Activation of the extrinsic pathway via treatment with TNFα or death-receptor ligand TRAIL resulted in rapid Annexin V labelling and cell death when co-treated with CHX, which prevents translation of pro-survival proteins (Figure 4a, black line; Supplementary Figure S2A). As a control to ensure Annexin V-positivity was the result of apoptosis, MEFs were co-treated with modulators of the apoptotic pathway, which caused the expected acceleration (ABT-737, an inhibitor of anti-apoptotic proteins) or inhibition of apoptosis (zVAD-fmk, a pan-caspase inhibitor), respectively (Figure 4a, red and brown lines).^18, 19^ Within these assays, YOYO3-positivity always followed Annexin V-positivity by a few hours and demonstrated a similar pattern of apoptotic kinetics (Figure 4b, Supplementary Figure S2B), and comparable staining efficiency in cells achieving late-stages of apoptosis (Figure 4c). Consistent with our previous results, similarly-treated cells analysed by flow cytometry failed to reveal differential staining between Annexin V and YOYO3, and we suspect the physical stress during processing promotes YOYO3 incorporation (Figure 4d). As a note, STS-treated cells demonstrated variable labelling by either Annexin V or YOYO3 (Figure 4e) and therefore use of TNFα and CHX or CHX and ABT are more suitable positive controls to measure maximal and accelerated cell death. Similar to the results obtained with death receptor ligands (Figures 4a–e), MEFs displayed similar kinetics of apoptosis when treated with inducers of UPR, ABT-737 and zVAD-fmk (Figures 4f and j).^20^ In these assays, however, the delay between Annexin V- and YOYO3-positivity was markedly pronounced compared to inducers of the extrinsic pathway (Figures 4h and i). Furthermore, different cell types demonstrate signature kinetic patterns between early and late apoptotic events (data not shown).

As high-throughput cytotoxic assays are frequently used for drug or RNAi screens, which engage the intrinsic pathway of apoptosis, these data suggest that the use of cell viability dyes in high-content cell imaging is imperfect due to an inaccurate and markedly delayed labelling of apoptotic populations. In contrast, the described Annexin V-labelling is highly sensitive, responsive and accurate for measuring the onset of apoptosis.

### Integrating cellular proliferation and apoptosis for accurate quantification of cell death

High-content live-cell imagers collect large quantities of cellular data by detecting fluorescent events in an imaging field. In contrast, flow cytometry-based Annexin V assays determine the number of positive cells within a population yielding a percentage (% Apoptosis). Data expressed as a percentage represent events normalized for total cell number, which often varies due to treatment-induced alterations in proliferation and growth arrest. As the requirement to define apoptosis as a percentage is critical, flow cytometry-based cell counting is often performed in tandem with high-content imaging analyses, negating the advantages of high-throughput technologies. Alternative and imperfect practices to circumvent this issue have been created, including: the staining of cellular populations with toxic DNA-intercalating dyes following the conclusion of high-content imaging data acquisition (Supplementary Figure S3A), or normalizing maximal apoptotic responses from events collected with potent apoptotic inducers such as STS or Actinomycin D.^21^ These methods fundamentally fail to account for dissimilarities of cell proliferation in response to treatments, the detachment of dead cells and undermine kinetic analyses by normalizing data to an end-point result. Furthermore, ‘maximal’ apoptotic responses by inducers such as STS can result in inconsistent labelling between cell types, and therefore normalizing to maximal inducers often obscures experimental interpretations.

**Figure 5:**
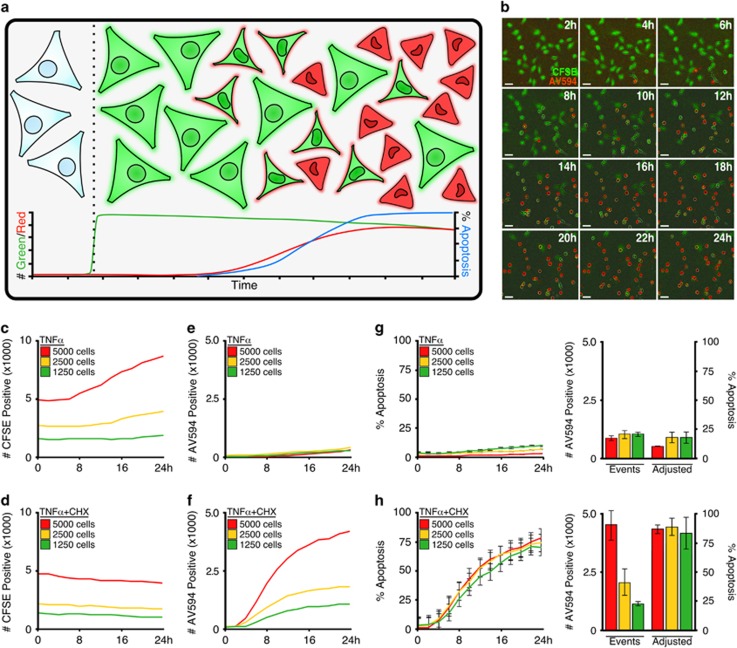
Integration of Annexin V and CFSE staining accurately detects treatment-induced changes in proliferation in populations undergoing apoptosis. (**a**) Schematic representation for the staining of cell population with CellTrace CFSE, incubating with Annexin V-594, inducing apoptosis, and analysing the number of labelled cells as a percentage. (**b**) MEFs were stained with CFSE (1 μM), and induced to undergo apoptosis with CHX (50 μg/ml) and ABT-737 (1 μM) in the presence of Annexin-594 (1 μg/ml). Images were collected by an IncuCyte ZOOM with a × 10 objective at indicated time points; 50 μm scale bar. (**c–f**) Indicated numbers of MEFs were plated, stained with CFSE, treated as indicated (TNF*α*, 10 ng/ml; CHX, 50 μg/ml), and incubated with Annexin V-594-containing culture media. CFSE-positive events shown in (**c** and **d**); Annexin V-positive events shown in (**e** and **f**). (**g**) Data shown in (**d** and **e**) were converted into % Apoptosis. (**h**) Data shown in (**d** and **f**) were converted into % Apoptosis. Bar graphs provide end-point data at 36 h following treatment. Error bars denote standard deviation

To address the above issues, we incorporated the highly stable, non-toxic, cell-permeable succinimydyl ester that targets intracellular lysines (for example, CellTrace CFSE) to label the total cellular population within each well (Supplementary Figure S3B). While traditionally used to characterize cell proliferation *in vivo*, carboxyfluorescein succinimidyl ester (CFSE) demonstrated potent and stable cellular labelling in culture (Supplementary Figure S3B, light green line). We therefore established a method in which total cells’ populations are stained with CFSE and coupled with Annexin V-labelling to accurately calculate the percent of the population succumbing to apoptosis in real time (Figure 5a).

Cells pre-labelled with CellTrace CFSE and then treated to undergo apoptosis in the presence of Annexin V-594 were readily detectable, and demonstrated a striking shift from green fluorescence to dual fluorescence upon apoptosis (Figure 5b). To validate the hypothesis that the CellTrace CFSE/Annexin V-594 combination allowed for the accurate quantification of total cells per well and apoptotic populations, we titrated the number of cells plated per well, treated with apoptotic stimuli and quantified the fluorescent events. CellTrace CFSE accurately measured the difference in cell number (Figures 5c and d), and accurately reported cell proliferation and no apoptosis (TNFα alone, Figures 5c and e). In contrast, cells dually treated with TNFα and CHX (revealing the pro-apoptotic function of TNFα signalling) demonstrated no proliferation (Figure 5d) and time-dependent Annexin V-staining (Figure 5f). Considering the minimal amount of CFSE photobleaching (Supplementary Figure S3B, light green line), data suggest the slight decrease in CFSE detection is the result of dead cell detachment into the supernatant, beyond the focal point of the imager. Therefore, CellTrace CFSE is a non-toxic, photostable, whole-population cellular stain that accurately measures changes in cell number resulting from changes in proliferation and loss of dead cells from detection.

Annexin V-positive data were converted to obtain a % Apoptosis utilizing a mathematical operation based on the real-time quantification of the CFSE whole-population. In most scenarios, % Apoptosis was attained by dividing Annexin V-positive events by CFSE-positive events. However, due to the specific detection parameters of high-content live-cell imagers, occasionally the mathematical conversion requires additional steps. In these instances, incorporation of a normalizing function is necessary (detailed equations are provided and explained within the Materials and Methods section). Using the above guidelines, data collected from treated cells (Figures 5c–f) were mathematically converted into a cell number-normalized representation of apoptotic kinetics expressed as an apoptotic percentage (Figures 5g and h, left panels). Furthermore, data collected by high-content kinetic detection of cells undergoing apoptosis is easily represented as a bar graph for easy communication of critical data (Figures 5g and h, right panels). Upon further examination, we found this method to accurately normalize apoptosis data in a number of kinetic profiles from controls (Supplementary Figures S4A–C), CHX (Supplementary Figures S4D–F), accelerated apoptosis via co-treatment with ABT-737 (Supplementary Figures S4G–I) or prevented apoptosis by co-treatment with zVAD-fmk (Supplementary Figures S4J–M). In response to the incomplete lack of apoptosis following co-treatment with zVAD-fmk, MEFs were also treated with Necrostatin-1, an inhibitor of programmed necrosis, to demonstrate that caspase inhibition did not result in activation of alternative cell death pathways (Supplementary Figures S4N–P).^22^

In combination with good cell culture practices, this mathematical conversion of detected events into percent apoptosis consistently acts as a control for cell number differences as a result of differential cell seeding, treatment-induced or cell type-inherent proliferation rates, and loss of detectible signal due to cellular detachment from the imager's focal plane. This adaptation of our described kinetic detection of apoptosis using real-time high-content cell imagers provides researchers with a quick and accurate method to assay experimental parameters in a high-throughput manner and represent data consistent with gold standard method in the field.

## Conclusion

In conclusion, here we describe a new method that couples recent high-content live-cell imaging technologies with classical Annexin V-binding approaches to accurately quantify the kinetics of apoptosis in single-cell and population studies. This new method demonstrates increased sensitivity and more rapid apoptotic reporting compared to current suggested protocols for high-throughput plate-based cell death studies. Furthermore, this versatile real-time method permits multiplex fluorescent detection with viability dyes to profile progression and detection of late events in apoptosis. Finally, experiments utilizing a non-toxic whole-population counter-stain demonstrated the capability to control for differences in cell number between samples due to cell proliferation and dead cell detachment from the imager’s focal plane. Collectively, this refined methodology provides a high-throughput, non-intensive, zero-handling, data-rich method to detect, quantify and visualize the kinetics of apoptosis.

## Materials and methods

### Reagents and equipment

Cell culture reagents and chemicals were from Sigma Aldrich (St. Louis, MO, USA) or Thermo Fisher Scientific (Waltham, MA, USA) unless otherwise stated. Drugs and biologics as follows: ABT-737 (Abbott Pharmaceuticals, Lake Bluff, IL, USA); Tn, Tg, CHX and STS (Sigma); DRAQ7 (Abcam, Cambridge, UK); YOYO3, CellTrace CFSE, DyeCycle Green and CellEvent Caspase-3/-7 Green Detection Reagent (Thermo Fisher Scientific); mTNFα and mTRAIL (Peprotech, Rocky Hill, NJ, USA); zVAD-fmk (ApexBio, Houston, TX, USA). Recombinant Annexin V was purified, labelled with FITC, DyLight 633 NHS ester (Thermo Fisher Scientific), or AlexaFluor 594 NHS ester (Thermo Fisher Scientific), and re-purified using Dye Removal Columns (Thermo Fisher Scientific) following conjugation. Purity, stability and fluorescence of labelled recombinant proteins were analysed by SDS-PAGE. Fluorescent imaging of labelled recombinant Annexin V was accomplished using an MP ChemiDoc Imager equipped with LED excitation cubes (Bio-Rad, Hercules, CA, USA).

### Tissue culture and cell preparation

Experiments were performed with SV40-transformed MEFs, U266, HeLa or primary MEFs where labelled. Cells were cultured in DMEM containing 10% FBS, 2 mM L-glutamine and antibiotics; U266 cells were cultured in RPMI 1640 containing 10% heat-inactivated FBS, and antibiotics. For kinetic studies, cells were seeded at approximately 3–5 × 10^3^ cells/well in a 96-well format or 8–10 × 10^3^ cells/well in a 48-well format for 24 h. Prior to live-cell imaging, media was replaced with phenol-red free complete DMEM containing: indicated treatments, DRAQ7 or YOYO3, and fluorescent Annexin V variants. For experiments utilizing DyeCycle (1 μg/ml in PBS) or CellTrace (1 μM in PBS), cells were incubated at 37 °C for 30 min before washing and replaced with the phenol-red free complete DMEM. Plates were pre-warmed prior to data acquisition to avoid condensation and expansion of the plate, which hinders autofocus. Experiments utilizing RPMI media, which contains 0.4 mM Ca^2+^, required CaCl_2_ supplementation for robust Annexin V labelling (data not shown).

### Flow cytometry

Cells were plated in 48-well plates at 1.5 × 10^4^, cultured overnight, and treated as described. At the time of collection, supernatants were collected and adherent cells were trypsinized for 5–10 min. Cells were pipetted up and down in trypsin to aid in detachment before combining with corresponding supernatant. Cells were pelleted via centrifugation at 1100 × *g* for 15 min and decanted. Unlike traditional Annexin V-binding protocols, cells were not resuspended in ABB but instead in complete DMEM to avoid cytotoxic effects of ABB. Staining was performed in DMEM containing Annexin V-488 (1 μg/ml) as well as YOYO3 (200 nM) for dual detection by flow cytometry.

### Data acquisition and analysis

Kinetic experiments were performed with the IncuCyte ZOOM (Essen Bioscience, Ann Arbor, MI, USA), but other high-content live-cell fluorescent imagers are capable of replicating these data. Experiments were conducted for 20–48 h with data collection every 2–4 h to avoid photobleaching of fluorescent reporters. Using the × 10 objective, four planes of view were collected per well for experiments using a 48-well plate; a single plane of view was collected for 96-well plate assays. Phase contrast, green channel (Ex: 440/80 nm; Em: 504/44 nm), and red channel (Ex: 565/05 nm; Em: 625/05 nm) were collected for all experiments with spectral unmixing set as 5% of red removed from green. Processing definitions are described below (Table 1). Data were always exported as events per well. Conversion of data to apoptotic percentages used the following algorithm (mathematically depicted below): raw percentages for each time point were calculated as Annexin V-positive events divided by the CellTrace value (Equation 1); an adjustment factor was calculated by dividing the difference between minimum and maximum percentages of all data points by 100 (Equation 2); each set of triplicate raw percentages was averaged and divided by the adjustment factor to obtain an adjusted percent from 0 to 100 inclusive (Equation 3).


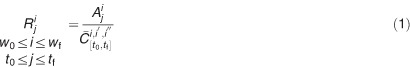



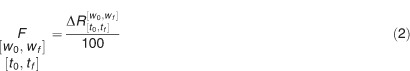



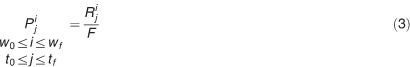


where: *R*=raw percentage*, F*=adjustment factor, *P*=adjusted percentage, *i*=arbitrary well, *j*=arbitrary time, *w*_*o*_=first well, *w*_f_ =last well, *t*_*o*_=initial time, *t*_f_ =final time, *A*=Annexin V value, 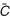
=average CellTrace value, *i/i’*/*i’’*=triplicate well set, [*w*_*o*_*,w*_f_]=inclusive range of well values, [*t*_*o*_,*t*_f_]=inclusive range of time data for wells.

## Figures and Tables

**Table 1:** Suggested processing definitions using the IncuCyte ZOOM

	*Parameter*	*Radius*	*Adjustment*	*Edge*	*Fill*	*Area*	*Eccentricity*	*Mean intensity*	*Integrated intensity*
Annexin V-FITC	Top-Hat	70	3.0	−30	0	0	0	>3	>500
Annexin V-FITC (puncta exclusion)	Top-Hat	25	3.0	−30	0	>100	0	0	0
DRAQ7	Adaptive	−	0.5	−48	5	>40	0	>1.5	0
YOYO3	Top-Hat	25	1.0	−46	0	>100	0	0	0
DEVD-green	Top-Hat	20	1.5	−37	0	>25	<0.9	0	0
Annexin V-594	Top-Hat	10	2	−35	0	>100	0	0	0
CellTrace	Top-Hat	25	0.5	−25	0	>150	0	>3	0

## Abbreviations

ABB: Annexin V binding buffer
BCL-2: B-cell lymphoma 2
CBB: Coomassie brilliant blue
CFSE: Carboxyfluorescein succinimidyl ester
CHX: cycloheximide
DMEM: Dulbecco’s modified Eagle’s medium
FBS: fetal bovine serum
FITC: Fluorescein isothiocyanate
MEF: mouse embryonic fibroblast
NHS Esters: N-hydroxysuccinimide esters
PARP: poly ADP ribose polymerase
PBS: phosphate-buffered saline
PI: propidium iodide
RPMI: Roswell Park Memorial Institute 1640
STS: staurosporine
Tg: thapsigargin
Tn: tunicamycin
TNF*α*: tumour necrosis factor *α*
TRAIL: TNF-related apoptosis-inducing ligand
UPR: unfolded protein response
zVAD-fmk: carbobenzoxy-valyl-alanyl-aspartyl-[O-methyl]-fluoromethylketone
