# Robust high-throughput kinetic analysis of apoptosis with real-time high-content live-cell imaging

**DOI:** 10.1038/cddis.2017.156

**Published:** 2017-05-04

**Authors:** Jesse D Gelles, Jerry Edward Chipuk

**Correction to:** *Cell Death and Disease* (2016) **7**, e2493; doi:10.1038/cddis.2016.332; published online 1 December 2016.

Since publication it has been noticed that an error during typesetting has led to the second author Jerry E Chipuk being incorrectly listed in PubMed as Edward Chipuk J. The publisher would like to apologise for any inconvenience caused.

