# Supplementary figures and images for: Robust high-throughput kinetic analysis of apoptosis with real-time high-content live-cell imaging
Source: Cell Death Dis. 2016 Dec 1;7(12):e2493–. doi: 10.1038/cddis.2016.332 (PMC5261025; doi:10.1038/cddis.2016.332)

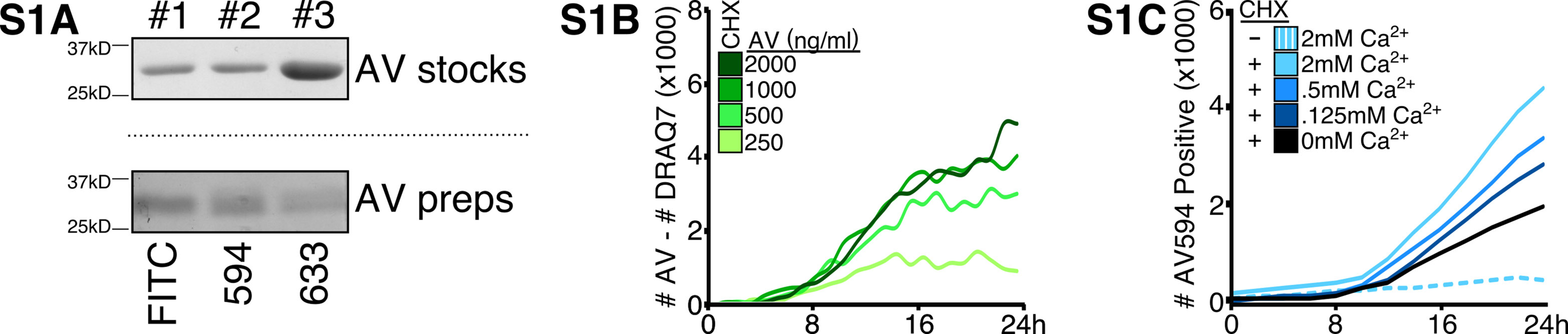

Supplement: Supplementary Figure 1 [file cddis2016332x1.tif]

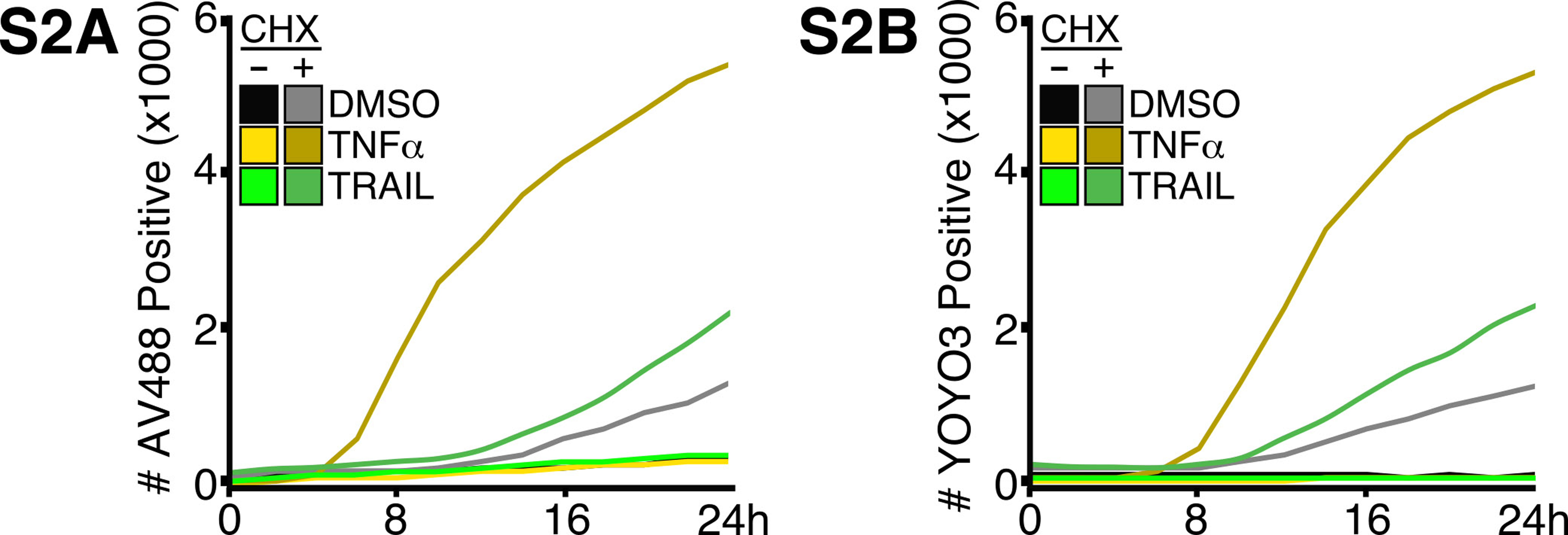

Supplement: Supplementary Figure 2 [file cddis2016332x2.tif]

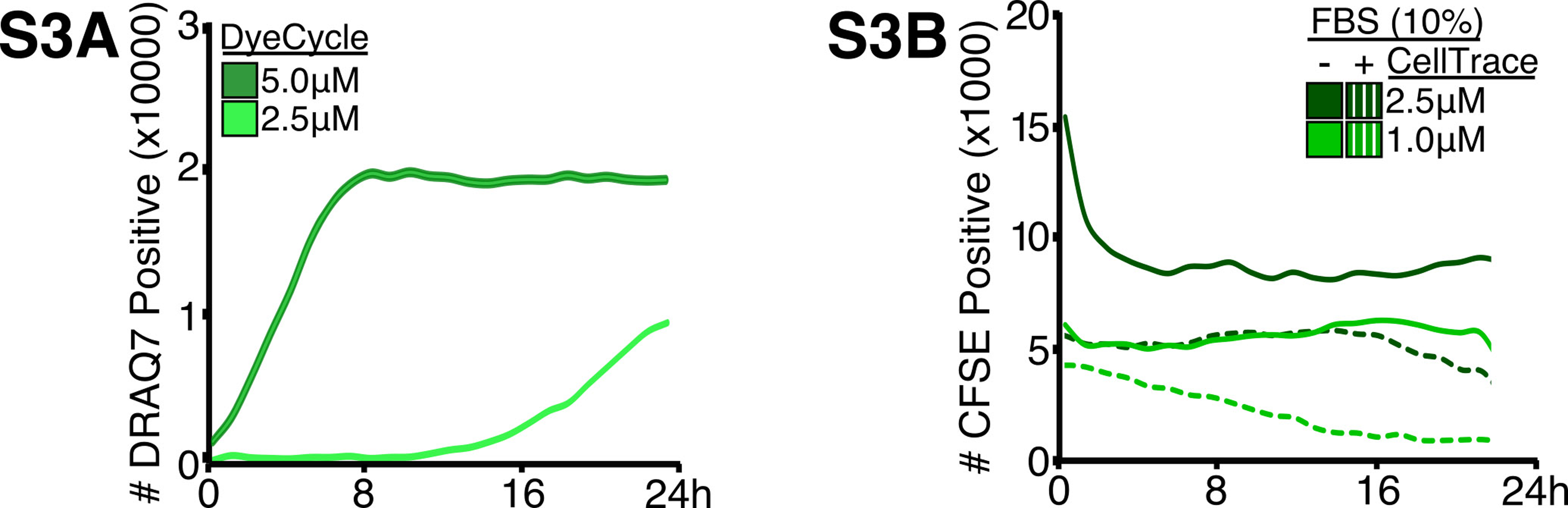

Supplement: Supplementary Figure 3 [file cddis2016332x3.tif]
